# Supplementary material for: Exploring the Association Between EEG Microstates During Resting-State and Error-Related Activity in Young Children
Source: Brain Topogr. 2023 Dec 23;37(4):552–70. doi: 10.1007/s10548-023-01030-2 (PMC11199242; doi:10.1007/s10548-023-01030-2)
Supplement: Supplementary file 1 — Supplementary Material 1: Supplementary Materials S1-S25 [file 10548_2023_1030_MOESM1_ESM.docx]

**SUPPLEMENTARY MATERIALS**

Table of Contents

[S1. Participant Eligibility & Recruitment 2](#_Toc145500748)

[S2. Correlations Between Neural Measures and Children’s Depressive Symptoms 4](#_Toc145500749)

[S3. Participant Demographics 5](#_Toc145500750)

[S4. Go/No-Go Task Visualization 6](#_Toc145500751)

[S5. EEG Preprocessing Steps 7](#_Toc145500752)

[S6. Go/No-Go Behavioral Data 10](#_Toc145500753)

[S7. Preprocessed EEG Data Quality Checks 11](#_Toc145500754)

[S8. Reliability of Neural Measures 12](#_Toc145500755)

[S9. Source Localization Additional Details 14](#_Toc145500756)

[S10. ERN Calculation (Unstandardized Residual Values) 15](#_Toc145500757)

[S11. Correlations Between Different ERN Scoring Procedures 16](#_Toc145500758)

[S12. Regression Analyses with Outliers Included in Models 17](#_Toc145500759)

[S13. Testing Ordinary Least Squares Regression Assumptions 24](#_Toc145500760)

[S14. Multiple Comparisons Correction 26](#_Toc145500761)

[S15. Cartool Output for Determining the Optimal Number of Clusters 27](#_Toc145500762)

[S16. Error-Related Microstate 3 Backfitting 28](#_Toc145500763)

[S17. Region-of-Interest for ERP Analyses 29](#_Toc145500764)

[S18. Polarity-Invariant Spatial Correlations Between Microstate Topographies 30](#_Toc145500765)

[S19. Descriptive Statistics of Microstate Temporal Parameters 31](#_Toc145500766)

[S20. Descriptive Statistics of Parent-Report Questionnaire Data 32](#_Toc145500767)

[S21. Four versus Six Microstate Resting-State Data Solutions 33](#_Toc145500768)

[S22. Full Details of Regression Analyses 35](#_Toc145500769)

[S23. Results from Models Including Resting-State Microstate 6 37](#_Toc145500770)

[S24. Neural Sources of Resting-State Microstate 6 and Error-Related Microstate 3 38](#_Toc145500771)

[S25. Overlap Between Resting-State Microstate 6 and Error-Related Microstate 3 Sources with Canonical Networks 39](#_Toc145500772)

## S1. Participant Eligibility & Recruitment

Inclusion & Exclusion Criteria

The following was copied from Bagdasarov et al. (2022) paper: “Participants were part of a larger study investigating reward processing in early emerging risk for depression. Parents were required to be the child’s biological mother and primary caregiver, and to have lived with the child for the last six months. Children were required to be 4-7 years old at the date of their first visit. Children were excluded if (1) they were born premature (< 35 weeks of gestation), (2) their biological mother drank alcohol, used illicit drugs, or smoked during pregnancy, (3) they ever experienced seizures, loss of consciousness, brain swelling, or hospitalization due to head trauma, (4) they were diagnosed with Hydrocephalus, Hypoxic-Ischemic Encephalopathy, Cerebral Palsy, a brain tumor, a genetic disorder, hearing or vision impairment, a chronic illness, Attention-Deficit/Hyperactivity Disorder, or a Pervasive Developmental Disorder (PDD), (5) they had delays in speech or language or other developmental delays, (6) they had a medical problem or illness that caused behavioral concerns, or (7) they took medications for mood, anxiety, or attention difficulties. Caregivers were also asked about their child's depressive symptoms using the short version of the Preschool Feelings Checklist (PFC-Short; Luby et al., 2004). Due to the primary aims of the overarching study, children with low (≤ 1 PFC items endorsed) or high (≥ 3 PFC items endorsed) levels of depressive symptoms were included while children with a moderate (2 PFC items endorsed) level were excluded.”

Recruitment

323: Screened

74: Ineligible

31: PFC-Short score of 2

7: Taking medication for ADHD

6: Diagnosed with ASD

30: Other or multiple criteria not met

249: Eligible

78: Did not complete Visit 1

17: Declined to participate

13: Cancelled due to illness (e.g., COVID-19)

48: Did not complete for other reason (e.g., did not show up to visit)

171: Completed visit 1, 63: Completed follow-up Visit 2 *

90: Had both resting-state & go/no-go EEG at visit 1 +

24: Had both resting-state & go/no-go EEG at visit 2

5 (of the above 90 + 24): Had both resting-state and go/no-go EEG at both visit 1 & 2

After EEG preprocessing, 20: Did not have usable data at visit 1 (i.e., useable = both clean resting-state and go/no-go EEG data)

7: Had bad resting-state EEG

11: Had bad go/no-go EEG

2: Had both bad resting-state & go/no-go EEG

3: Did not have usable data at visit 2

1: Had bad resting-state EEG

1: Had bad go/no-go EEG

1: Had both bad resting-state & go/no-go EEG

1: Had good resting-state & go/no-go EEG at both visit 1 & 2; thus, only their visit 1 data was used

**Final sample = 90 participants**

**Data for final sample collected April 2019 – June 2021**

** Participant retention for follow-up visit 2 was low largely due to the COVID-19 pandemic. Further, resting-state EEG was not part of the original protocol; it was added halfway through data collection, and as a result, the number of participants who had both resting-state and task EEG was much lower than the number of participants (171) who completed visit 1.*

Bagdasarov, A., Roberts, K., Bréchet, L., Brunet, D., Michel, C. M., & Gaffrey, M. S. (2022). Spatiotemporal dynamics of EEG microstates in four-to eight-year-old children: Age-and sex-related effects. *Developmental cognitive neuroscience*, *57*, 101134.

## S2. Correlations Between Neural Measures and Children’s Depressive Symptoms

The primary aim of the overarching study was to investigate reward processing in early emerging risk for depression. Therefore, we assessed Pearson’s correlations between EEG measures and children’s depressive symptoms. We found no statistically significant correlations (see below). As such, we did not control for children’s depressive symptoms in statistical models.

|  | Correlation (*r*) | *p* value |
| --- | --- | --- |
| Resting-State Microstate 4 GEV | .05 | .66 |
| Resting-State Microstate 6 GEV | .02 | .86 |
| Error-Related Microstate 3 GEV | .11 | .31 |
| Residualized ERN | -.16 | .15 |

*Note.* Children’s depressive symptoms are represented as the unstandardized residual values from a regression model in which the Beck Depression Inventory, 2nd edition (BDI-II; Beck et al., 1996) was entered as the independent variable and the Preschool Feelings Checklist, Scale Version (PFC-Scale; Luby et al., 2012) was entered as the dependent variable (these measures were collected at each study visit as part of the overarching study). This procedure reduced the inflation of children’s depressive symptoms by maternal depressive symptoms. The sample size for this analysis was reduced to 80 because 6 participants did not have error-related microstate 3 and an additional 4 participants did not have a PFC-Scale and/or BDI-2 score.

## S3. Participant Demographics

Reduced sample excludes participants without error-related microstate 3 (*n* = 84).

|  | *n* = 84 | | *n* = 90 | | Comparison |
| --- | --- | --- | --- | --- | --- |
|  | Mean (SD) | Range | Mean (SD) | Range | *p* value ^+^ |
| Age (years) | 6.52 (1.09) | 4.51 – 8.96 | 6.49 (1.10) | 4.51 – 8.96 | .86^A^ |
| Income-to-Needs Ratio * | 3.01 (1.00) | 0.69 – 4.69 | 3.06 (1.03) | 0.69 – 4.69 | .76^A^ |
|  | *n* | Percent (%) | *n* | Percent (%) |  |
| Biological Sex |  |  |  |  | .93^B^ |
| Females | 47 | 55.95 | 52 | 57.78 |  |
| Males | 37 | 44.05 | 38 | 42.22 |  |
| Race |  |  |  |  | .99^C^ |
| White | 62 | 73.81 | 65 | 72.22 |  |
| Multiracial | 12 | 14.29 | 14 | 15.56 |  |
| Black or African American | 6 | 7.14 | 7 | 7.78 |  |
| Asian | 2 | 2.38 | 2 | 2.22 |  |
| Other | 2 | 2.38 | 2 | 2.22 |  |
| Native Hawaiian or Other Pacific Islander | 0 | 0 | 0 | 0 |  |
| Ethnicity |  |  |  |  | >.99^B^ |
| Not Hispanic or Latino | 72 | 85.71 | 78 | 86.67 |  |
| Hispanic or Latino | 12 | 14.29 | 12 | 13.33 |  |
| Maternal Education |  |  |  |  | >.99^C^ |
| Some Grade School | 0 | 0 | 0 | 0 |  |
| Completed Grade School | 0 | 0 | 0 | 0 |  |
| Some High School | 0 | 0 | 0 | 0 |  |
| High School Diploma | 1 | 1.19 | 1 | 1.11 |  |
| Some College or 2-Year Degree | 19 | 22.62 | 20 | 22.22 |  |
| 4-Year College Degree | 25 | 29.76 | 27 | 30 |  |
| Some School Beyond College | 1 | 1.19 | 1 | 1.11 |  |
| Professional or Graduate Degree | 38 | 45.24 | 41 | 45.56 |  |

*Note.* *The Income-to-Needs Ratio (ITN) was calculated by dividing total family income by a poverty threshold determined by the United States Census Bureau, which considered the year assessed and household family size. One participant who is in both samples did not have ITN information available. ^+^Statistical analyses were performed to determine whether there were significant differences in demographic characteristics between groups. ^A^Welch’s *t*-test. ^B^Chi-squared test. ^C^Fisher’s exact test. Results revealed no significant differences in demographics characteristics between groups.

## S4. Go/No-Go Task Visualization

## S5. EEG Preprocessing Steps

| **Step** | **Method** | **Resting-State Only Notes** | **Go/No-Go Only Notes** | **Overall Notes** |
| --- | --- | --- | --- | --- |
| 1 | Remove outer ring of channels. |  |  | 24 channels removed because they contained a large amount of artifact given their location near the base of the skull or on the neck or face. |
| 2 | Downsample from 1000 Hz to 250 Hz. |  |  |  |
| 3 | Remove data during breaks. | Markers to signify eyes- open versus closed data were added, and eyes-open data were removed as well. |  |  |
| 4 | Filter. | 1 to 40 Hz bandpass. | 0.1 to 40 Hz bandpass. | Hamming windowed sinc finite impulse response (FIR) filter. |
| 5 | Remove 60 Hz line noise. |  |  | Despite the 40 Hz low-pass filter, electrical line noise was still present for some participants and subsequently attenuated using CleanLine (Mullen, 2012). |
| 6 | Remove bad channels. |  |  | Criteria: 1) flat for more than 5 seconds, 2) contained more than 4 standard deviations of line noise relative to its signal (based on the total channel population), or 3) correlated at less than .8 to an estimate based on nearby channels. |
| 7 | Interpolate bad channels. |  |  | Spherical spline interpolation. |
| 8 | Re-reference to the average. |  |  |  |
| 9 | Create a copy of the data, remove artifacted portions with Artifact Subspace Reconstruction (ASR), and re-reference the data again. |  | Before ASR, 1 Hz high-pass filter applied to a copy of the data. | ASR: Burst criterion or maximum acceptable 0.5 second window standard deviation set conservatively to 20 (Chang et al., 2018). Data were removed, not reconstructed, and on a copy of the data.  Additional periods of data marked as bad if a channel’s power exceeded 7 standard deviations; periods removed only if more than 25% of channels during a given period were marked as bad. |
| 10 | Perform ICA with PCA dimension reduction on data resulting from previous step and flag components related to eye or muscle artifacts. | 30 PCA components | 50 PCA components | Extended infomax ICA with PCA dimension reduction given large number of channels and relatively small number of data points. More continuous ERP data than resting-state data = more PCA components for ERP data.  ICLabel to flag components with >= 70% of being related to eye or muscle artifacts. |
| 11 | Copy ICA fields to full-length data and remove flagged components. |  |  | Full-length data = data just before ASR removed portions containing artifacts. All following steps are performed on this full-length data set. In this way, ASR was only used to feed ICA clean data to maximize its performance. |
| 12 | Segment data. | Create nonoverlapping 1-second epochs. | Performed in ERPLAB. Load event list and bins file. Extract bin-based epochs -500 to 800 ms relative to button-press. Apply baseline correction during -500 to -300 ms period. |  |
| 13 | Epoch-level artifact rejection, epoch-level channel interpolation, and removal of epochs based on reaction times. | Epochs were removed if they had: At least 10 channels with 1) amplitudes greater than 100 μV or less than -100 μV, or 2) joint probabilities (i.e., probabilities of activity) above 3 standard deviations for local or global thresholds. If less than 10 channels met rejection criteria, the epoch was not removed, but the channels were interpolated for that epoch only. | Epochs were removed if they had: At least 10 channels with 1) peak-to-peak amplitudes exceeding 100 μV within 200 ms windows sliding by 20 ms, 2) amplitudes greater than 150 μV or less than -150 μV, or 3) joint probabilities (i.e., probabilities of activity) above 3 standard deviations for local or global thresholds. If less than 10 channels met rejection criteria, the epoch was not removed, but the channels were interpolated for that epoch only.  Remove epochs if button was pressed less than 100 ms after stimulus presentation (too fast) or more than 2000 ms after stimulus presentation (too slow). | TBT plugin (Ben-Shachar, 2018) used for epoch-level artifact rejection and epoch-level channel interpolation. Custom scripts used for removal of epochs based on reaction times for task data only. |

Additional details are provided in Matlab scripts available on GitHub.

Software and Tools Versions

| **Software / Tool** | **Version** |
| --- | --- |
| EEGLAB | 2021.0 |
| CleanLine | 2.00 |
| ERPLAB | 9.00 |
| ICLabel | 1.4 |
| TBT | 2.6.1 |
| clean_rawdata | 2.7 |
| firfilt | 2.6 |
| CARTOOL | 4.09 (7498) |

## S6. Go/No-Go Behavioral Data

The mean accuracy (i.e., responding correctly by pressing the button for aliens and withholding button-press for astronauts) was .68 (standard deviation = .14, min = .23, max = .91). The mean reaction time for correct “go” trials was 545.04 ms (standard deviation = 91.32 ms). The mean reaction time for erroneous “no-go” trials was slightly lower at 499.41 ms (standard deviation = 120.61 ms).

## S7. Preprocessed EEG Data Quality Checks

Participants were only included in analyses if they met quality checks.

Criteria for both resting-state and go/no-go data:

1. Bad Channels
   - Number of bad channels must not exceed 15 (approximately 15% of 105 total channels).
   - Visualization of bad channels must not show clusters of bad channels.
2. Artifact Subspace Reconstruction (ASR)
   - Data length after ASR must exceed 60 seconds.
3. Independent Component Analysis (ICA)
   - Visualization of decomposition must appear “normal” and “appropriate.”
   - Visualization of flagged components must appear eye- or muscle-related.
   - Retained variance after removal of flagged components must exceed 50%.
4. Channel Power Spectra
   - Visualization of channel power spectra must appear “normal” and “appropriate.”

For resting-state data:

1. At least 120 seconds of data must be available.

For go/no-go data:

1. At least 6 trials for both Correct and Error bins.

Descriptive statistics of resting-state EEG data quality metrics.

|  | Number of Channels Removed and Interpolated | File Length in Seconds After ASR | Number of Independent Components Rejected | Percent Variance Retained After Removal of Independent Components | File Length in Seconds After Preprocessing |
| --- | --- | --- | --- | --- | --- |
| Mean | 4.29 | 238.36 | 2.68 | 77.10 | 217.09 |
| SD | 3.15 | 37.81 | 1.20 | 11.88 | 12.86 |
| Minimum | 0 | 82.69 | 0 | 50.02 | 157 |
| Maximum | 13 | 238.36 | 7 | 100 | 234 |

*Note.* Descriptive statistics are for the 90 clean EEG files. ASR = Artifact Subspace Reconstruction.

Descriptive statistics of go/no-go EEG data quality metrics.

|  | Number of Channels Removed and Interpolated | File Length in Seconds After ASR | Number of Independent Components Rejected | Percent Variance Retained After Removal of Independent Components | Number of Correct Trials After Preprocessing | Number of Error Trials After Preprocessing |
| --- | --- | --- | --- | --- | --- | --- |
| Mean | 4.43 | 350.10 | 3.42 | 83.07 | 87.96 | 20.53 |
| SD | 3.64 | 56.07 | 2.46 | 10.15 | 21.03 | 9.98 ` |
| Minimum | 0 | 171.19 | 0 | 54.07 | 21 | 6 |
| Maximum | 13 | 421.77 | 12 | 100 | 114 | 47 |

*Note.* Descriptive statistics are for the 90 clean EEG files. ASR = Artifact Subspace Reconstruction.

## S8. Reliability of Neural Measures

Odd-Even Split-Half Reliability Resting-State Microstates

The internal consistency of resting-state data was calculated with odd-even split-half reliability. Each participant’s data was split into 6 equal, 26-second epochs representing the first 156 seconds of data (the last second was discarded so epochs were of equal duration). Epochs were combined in an odd-even fashion, resulting in 2, 78-second sets of data for each participant. Backfitting as described in the Methods section was performed for each of the two sets separately. The Spearman-Brown Prophecy Formula was used to calculate the split-half correlation for GEV values of microstates 4 and 6 (Brown, 1910; Spearman, 1910). The internal consistency of microstate 4 GEV was .97. The internal consistency of microstate 6 GEV was also .97. These values were identical when calculated with either the full sample of 90 participants or the reduced sample of 84 participants (i.e., excluding participants without error-related microstate 3).

Odd-Even Split-Half Reliability of Error-Related Microstate 3

The internal consistency of error-related microstate 3 was calculated with odd-even split-half reliability. For each participant, the averaged activity of odd correct “go” trials was subtracted from the averaged activity of odd erroneous “no-go” trials. Similarly, the averaged activity of even correct “go” trials were subtracted from the averaged activity of even erroneous “no-go” trials. This procedure created two sets of error-related activity for each participant. Backfitting as described in the Methods section was performed for each of the two sets separately. The Spearman-Brown Prophecy Formula was used to calculate the split-half correlation for GEV values of error-related microstate 3, which was .45 (Brown, 1910; Spearman, 1910). Prior work has demonstrated the reliability of subtraction-based activity (e.g., error minus correct) to be inherently lower than the activity of individual trial types (e.g., correct versus error), particularly when individual trial types are highly correlated (Clayson et al., 2021). In fact, above we showed a significant correlation between trial types at the amplitude-level when calculating residualized ERN values. Further, splitting trials in half results in noisier microstate topographies (i.e., they are less smooth) at the participant-level and may decreased reliability.

Dependability of the ERN

The internal consistency of the ERN was calculated using the ERP Reliability Analysis Toolbox with default parameters (Clayson & Miller, 2017), except for the number of cores which was set to 4 and the number of iterations which was set to 10,000. Internal consistency was measured by the index of dependability (Clayson & Miller, 2017). The tables below provide the index of dependability with confidence intervals for correct “go” trials and erroneous “no-go” trials as well as the mean, standard deviation, and range of the number of trials across participants. Notably, and as expected, participants had many more correct “go” trials than erroneous “no-go” trials, thereby increasing reliability of correct “go” trials. According to Clayson & Miller (2017), the research decides the acceptable level of score reliability. They also suggest values of .70 or .80 may be appropriate. As such, the dependability values below were determined to be sufficiently appropriate.

See here for toolbox and more information on the index of dependability: <https://github.com/peclayson/ERA_Toolbox>

*n* = 90 (i.e., all participants)

*n* = 84 (i.e., excluding participants without error-related microstate 3)

*Note*. CI = Confidence Interval. LL = Lower Limit. UL = Upper Limit. SD = Standard Deviation.

Brown, W. (1910). Some experimental results in the correlation of mental abilities 1. *British Journal of Psychology, 1904‐1920*, *3*(3), 296-322.

Clayson, P. E., & Miller, G. A. (2017). ERP Reliability Analysis (ERA) Toolbox: An open-source toolbox for analyzing the reliability of event-related brain potentials. *International Journal of Psychophysiology*, *111*, 68-79.

Clayson, P. E., Baldwin, S. A., & Larson, M. J. (2021). Evaluating the internal consistency of subtraction‐based and residualized difference scores: Considerations for psychometric reliability analyses of event‐related potentials. *Psychophysiology*, *58*(4), e13762.

Spearman, C. (1910). Correlation calculated from faulty data. *British journal of psychology*, *3*(3), 271.

## S9. Source Localization Additional Details

- The MRI template was an average of 82, 4.5- to 8.5-year-old children from the National Institute of Health (NIH) funded MRI Study of Normal Brain Development (Fonov et al., 2011).
- The 4-shell model represented the skull thickness and conductivity of a 6.5-year-old child (the rounded up mean age of participants in the current study), incorporating the scalp, skull, cerebrospinal fluid, and brain, and offering exact solutions for any number of isotropic spherical layers.
- Source maps were converted to volumes by computing all intermediate voxels from the grey matter-constrained head model using cubic splines. No new maxima were artificially created.
- AFNI was used to identify activated sources with the Talairach-Tournoux Atlas.

Fonov, V., Evans, A. C., Botteron, K., Almli, C. R., McKinstry, R. C., Collins, D. L., & Brain Development Cooperative Group. (2011). Unbiased average age-appropriate atlases for pediatric studies. *Neuroimage*, *54*(1), 313-327.

## S10. ERN Calculation (Unstandardized Residual Values)

Linear regression results revealed that during the -64 to 108 ms period, the correct “go” ERP mean amplitude explained a significant proportion of the observed variation in the erroneous “no-go” ERP mean amplitude, adjusted *R*^2^ = .31, *F*(1, 88) = 41.63, *p* < .001. A larger correct “go” ERP mean amplitude was related to a larger erroneous “no-go” ERP mean amplitude, *t*(88) = 0.67, *p* < .001, 95% CI [0.46, 0.87]. Unstandardized residual values from this model were extracted for each participant.

## S11. Correlations Between Different ERN Scoring Procedures

|  | M1 Diff | M1 Resid | M2 Diff | M2 Resid |
| --- | --- | --- | --- | --- |
| M1 Diff | - |  |  |  |
| M1 Resid | .95 | - |  |  |
| M2 Diff | .95 | .89 | - |  |
| M2 Resid | .87 | .93 | .93 | - |

- M1 (Method 1): Mean amplitude during the microstate-defined -64 to 108 ms period
  - This is the method used for all analyses in the current paper
- M2 (Method 2): Mean amplitude of a 96 ms period surrounding the microstate-defined -64 to 108 ms period for each participant (i.e., 48 ms on each side)
- Diff (Difference Score): Error minus correct trials
- Resid (Residualized Score): Unstandardized residual values (procedure described previously)

## S12. Regression Analyses with Outliers Included in Models

Model 1: No outliers

Model 2: 2 outliers

Step 1:


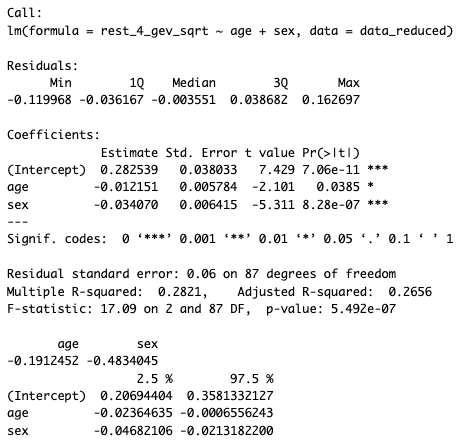


Step 2:


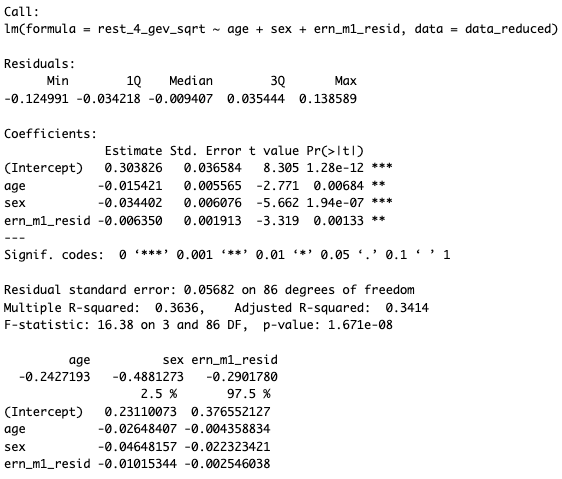


ANOVA to Compare Steps:


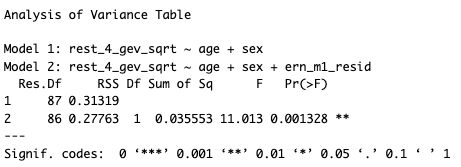


Partial *R*^2^:


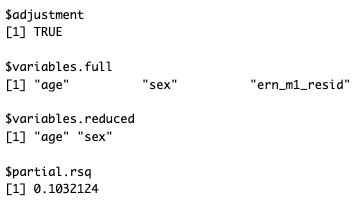


Model 3: 2 outliers

Step 1:


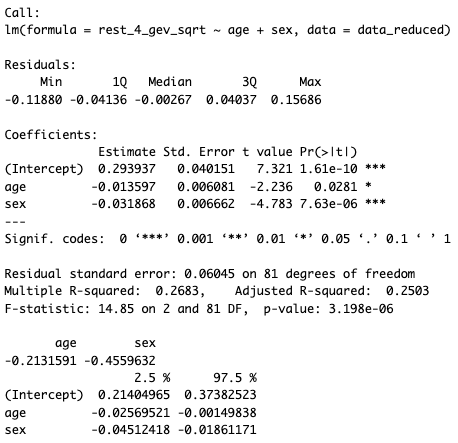


Step 2:


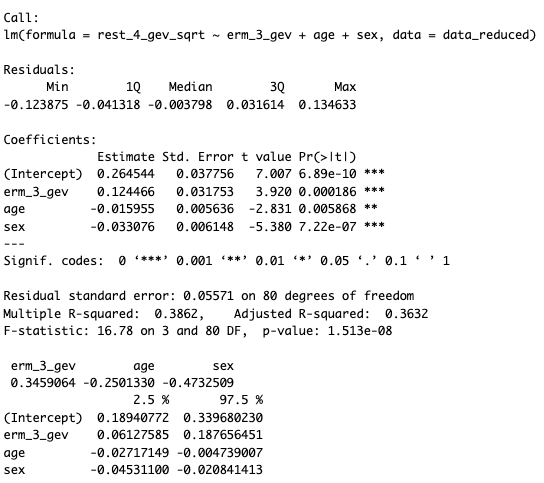


ANOVA to Compare Steps:


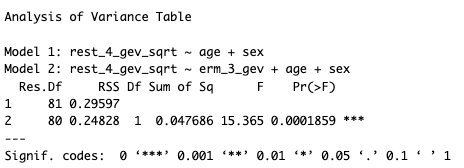


Partial *R*^2^:


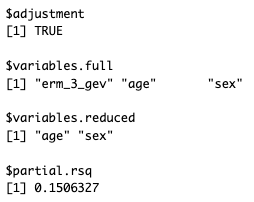


Model 4: 3 outliers

Step 1:


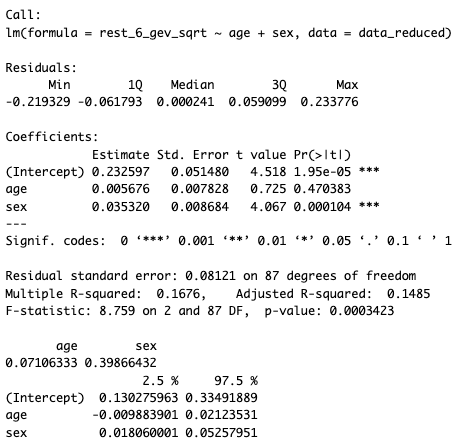


Step 2:


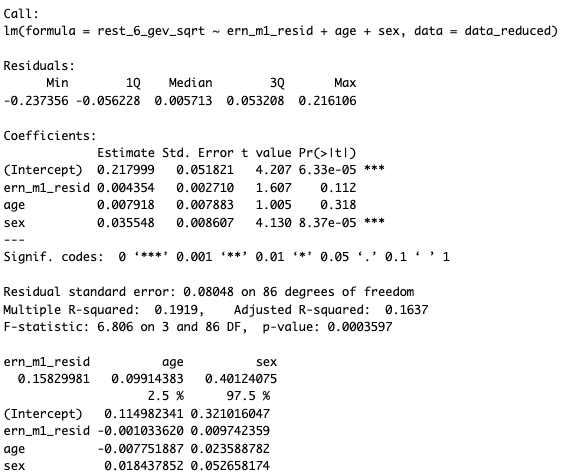


ANOVA to Compare Steps:


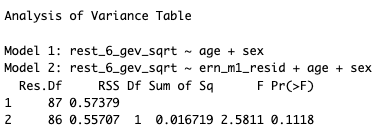


Partial *R*^2^:


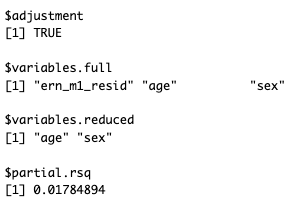


Model 5: 2 outliers

Step 1:


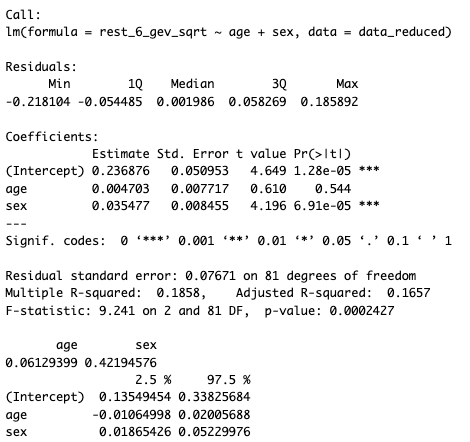


Step 2:


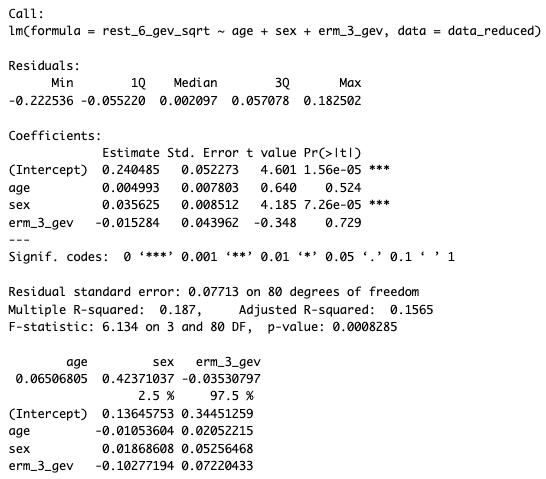


ANOVA to Compare Steps:


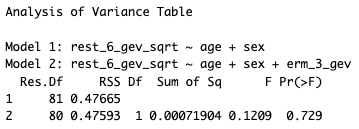


Partial *R*^2^:


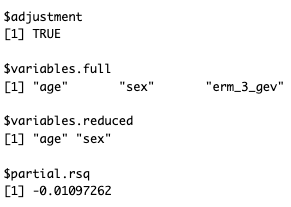


Model 6: 1 outlier


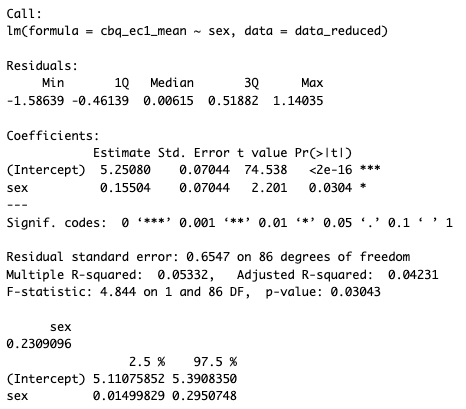


Model 7: 4 outliers


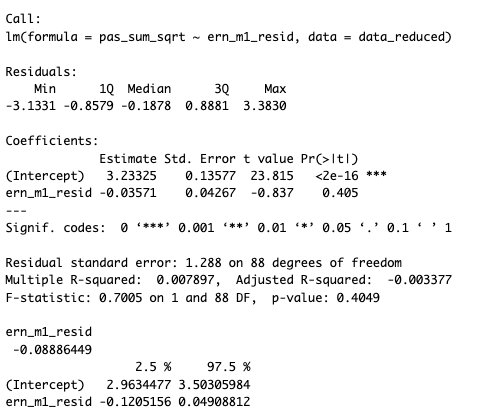


Model 8: 2 outliers

Step 1:


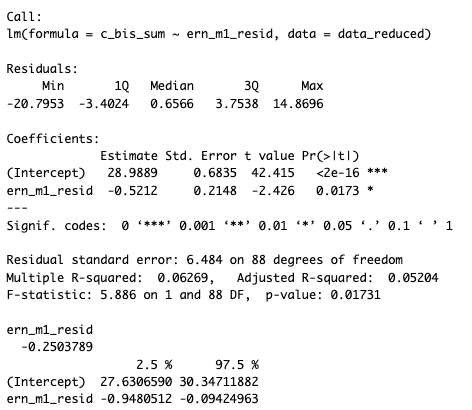


Step 2:


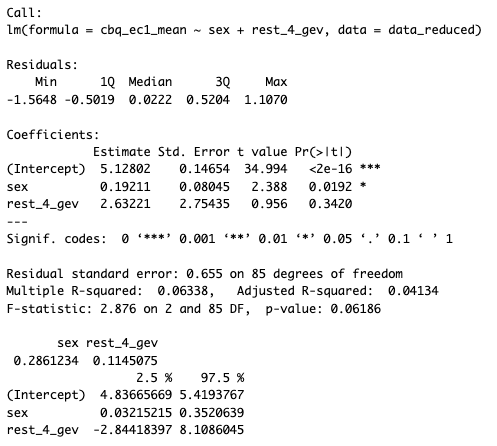


ANOVA to Compare Steps:


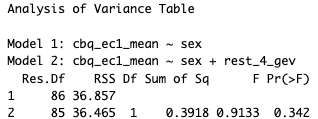


## S13. Testing Ordinary Least Squares Regression Assumptions

Gauss-Markov Assumptions

1. The population model is linear in its parameters.
2. A simple random sample has been drawn from a single underlying population.
   - No outliers: Outliers should be identified and removed. We use the MCD.
3. The conditional mean other-factor score is zero.
   - Should be no correlation between allowed-for factor variances and other-factor scores.
4. Absence of perfect collinearity.
   - Predicts should not be highly correlated.
   - Variance inflation factor (VIF): > 4 warrants investigation while > 10 needs correction.
   - Condition Index: > 15 warrants investigation while > 30 needs correction.
5. Homoscedastic errors (i.e., homogeneity of variance).
   - Residuals versus fitted values scatterplot: Points should be spread evenly around the line (i.e., no funneling).
   - Breusch-Pagan test: The *p*-value should be > .05.
6. Absence of serial correlation of other-factor scores (i.e., independent errors).
   - Durbin-Watson test: Value should be between 1 and 3.

Important Additional Assumption:

1. Normality of other-factor scores (i.e., normally distributed errors).
   - Normal probability plot (residual Q-Q plot): Points should lie along the line.
   - Residuals histogram: Distribution of residuals should be roughly a bell-curve. This does NOT refer to predictors, only the errors.
   - Shapiro-Wilk test: The *p*-value should be > .05.

Corrective Actions for Failed Assumptions

- Transformation of the independent variable is often considered first to address nonlinearity.
- Transformation of the dependent variable should be considered to address heteroscedasticity and nonnormality.
- For a dependent variable with a positive skew, square root or natural log transformation may be best.
- For a dependent variable with a negative skew, raising scores to a power greater than one may be best.

Assumptions of Current Models

|  | Model 1 | Model 2 | Model 3 | Model 4 | Model 5 | Model 6 | Model 7 | Model 8 |
| --- | --- | --- | --- | --- | --- | --- | --- | --- |
| Assumption 1 |  |  |  |  |  | NA | NA | NA |
| Assumption 2 |  |  |  |  |  |  |  |  |
| Assumption 3 |  |  |  |  |  |  |  |  |
| Assumption 4 |  |  |  |  |  |  |  |  |
| Assumption 5 |  |  |  |  |  |  |  |  |
| Assumption 6 |  |  |  |  |  |  |  |  |
| Assumption 7 |  |  |  |  |  |  |  |  |

Green = Assumption is met

Yellow = Assumption is not met & needs corrective actions

Corrective Actions Taken *

- Model 1: Square root transformed dependent variable.
- Model 2: Square root transformed dependent variable.
- Model 3: Square root transformed dependent variable.
- Model 4: Square root transformed dependent variable.
- Model 5: Square root transformed dependent variable.
- Model 6: Square root transformed dependent variable.
- Model 7: None. Not needed.
- Model 8: None. Not needed.

* After taking corrective actions, models were re-run. All assumptions were met.

## S14. Multiple Comparisons Correction

| Model | Original *p*-value | Corrected *p*-value |
| --- | --- | --- |
| 1 | .000000004998 | 0.00000001333 |
| 2 | .000000002448 | 0.00000001333 |
| 3 | .000000004702 | 0.00000001333 |
| 4 | .004008 | 0.00534400000 |
| 5 | .002399 | 0.00383840000 |
| 6 | .9899 | 0.98990000000 |
| 7 | .001643 | 0.00328600000 |
| 8 | .1202 | 0.13737142857 |

## S15. Cartool Output for Determining the Optimal Number of Clusters

## S16. Error-Related Microstate 3 Backfitting

Percentage of participants with backfitted error-related microstate 3 present at each timepoint of error-related activity.


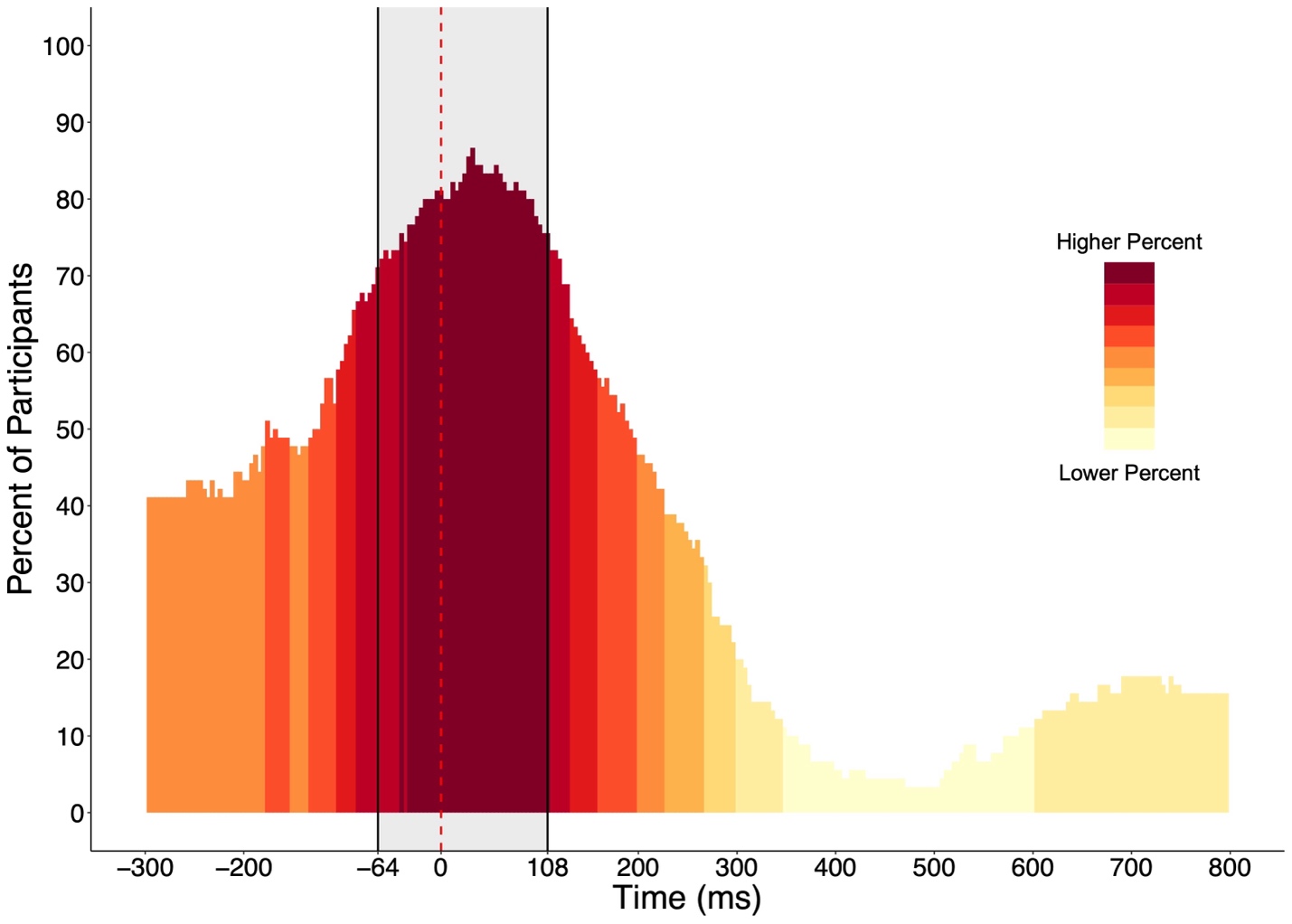


*Note.* The dashed red line represents the time of button-press. The highlighted shaded grey area represents the time period of error related microstate 3. Importantly, this figure was made by backfitting error-related microstate 3 to the entire segment of error-related activity (-300 to 800 ms) for visualization purposes only. That is, participants’ GEV values were derived only when backfitting error-related microstate 3 to the -64 to 108 ms period (not to the entire -300 to 800 ms period as done here for visualization purposes).

Descriptive Statistics of the Latency of Onset and Duration of Error-Related Microstate 3

|  | Mean | Standard Deviation | Min | Max |
| --- | --- | --- | --- | --- |
| Onset Latency | -53.33 ms | 25.36 ms | -64 ms | 32 ms |
| Mean Duration | 148.40 ms | 42.14 ms | 39.80 ms | 176 ms |

## S17. Region-of-Interest for ERP Analyses

128-Channel HydroCel Geodesic Sensory Net


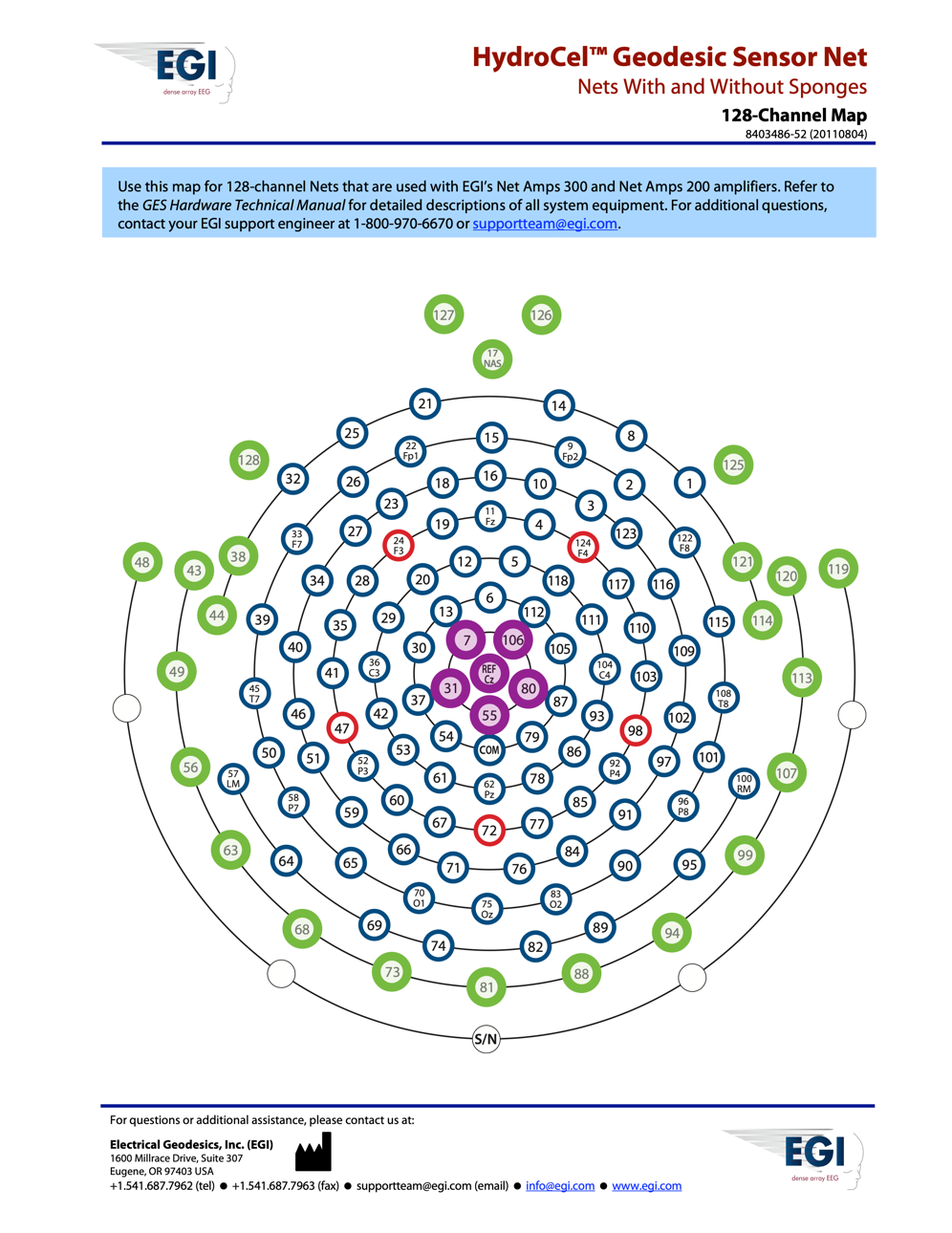


**Green** channels represent the 24 typically noisy outer ring that were removed during preprocessing. **Purple** channels represent the 6 channels used for ERN.

## S18. Polarity-Invariant Spatial Correlations Between Microstate Topographies

## S19. Descriptive Statistics of Microstate Temporal Parameters

Error-Related Microstates

- Group-level
  - GEV of the 10-map solution for the entirety of error-related activity = 96.97%
  - GEV of error-related microstate 3 = 26% (highest GEV of all maps in 10-map solution)
- Individual-level
  - Unlabeled time-frames of backfitting results (i.e., backfitting of error-related microstate 3 during the -64 to 108 ms (44 time-frames total for each participant) period):
    - Mean = 18.91%
    - Standard deviation = 29.10%
    - After excluding the six participants who do not have error-related microstate 3:
      - Mean = 13.19%
      - Standard deviation = 19.86%
  - GEV
    - Mean = .36
    - Standard deviation = .19

Resting-State Microstates

- Group-level
  - GEV of the six microstates = 82%
- Individual-level
  - Unlabeled time-frames of backfitting results (i.e., backfitting of all six microstates, 39,250 time-frames total for each participant):
    - Mean = 1.71%
    - Standard deviation = 0.91%
  - Microstate 4 GEV
    - Mean = .04
    - Standard deviation = .03
  - Microstate 6 GEV
    - Mean = .08
    - Standard deviation = .05

## S20. Descriptive Statistics of Parent-Report Questionnaire Data

CBQ-SF Effortful Control (*n* = 88*)

- Mean = 5.27
- Standard Deviation = 0.67
- Range = 3.65-6.47

* Sample is reduced by two participants because one participant had too many “prefer not to answer” responses and one participant was missing the measure entirely.

PAS (*n* = 90)

- Mean = 12.09
- Standard Deviation = 8.97
- Range = 0-43

BIS Scale (*n* = 90)

- Mean = 28.99
- Standard Deviation = 6.66
- Range = 9-43

## S21. Four versus Six Microstate Resting-State Data Solutions

There is an ongoing debate for how to select the optimal number of resting-state microstates for a given dataset. The current literature suggests, among others, two methods: 1) forcing a four-microstate solution based on previous work, or 2) using a meta-criterion as provided e.g., by Cartool software (Bréchet et al., 2019; Custo et al., 2017). Below, we show that the topography and temporal parameters of microstate 4 are highly similar regardless of the method used to select the optimal number of microstates. That is, our results showing relationships between microstate 4 and other variables (e.g., error-relate microstate 3) are highly unlikely to change whether using a four- or six-microstate solution.

Topographies (polarity-invariant)

Spatial Correlations (polarity-invariant)

|  | Microstate 1^b^ | Microstate 2 ^b^ | Microstate 3 ^b^ | Microstate 4 ^b^ | Microstate 5 ^b^ | Microstate 6 ^b^ |
| --- | --- | --- | --- | --- | --- | --- |
| Microstate 1^a^ | .89 | .40 | .35 | .09 | .93 | .49 |
| Microstate 2^a^ | .40 | >.99 | .68 | .42 | .73 | .41 |
| Microstate 3^a^ | .81 | .58 | .97 | .47 | .17 | .86 |
| Microstate 4^a^ | .46 | .33 | .58 | >.99 | .02 | .07 |

*Note*. ^a^ Four-microstate solution; ^b^ six-microstate solution.

Global Explained Variance (GEV) Pearson’s Correlations

|  | Microstate 1^b^ | Microstate 2 ^b^ | Microstate 3 ^b^ | Microstate 4 ^b^ | Microstate 5 ^b^ | Microstate 6 ^b^ |
| --- | --- | --- | --- | --- | --- | --- |
| Microstate 1^a^ | .54** | -.01 | -.63** | -.15 | .80** | .20 |
| Microstate 2^a^ | -.57** | .99** | -.19 | -.18 | .45** | .06 |
| Microstate 3^a^ | .13 | -.35** | .75** | -.53** | -.61** | .02 |
| Microstate 4^a^ | .01 | -.27* | -.16 | .99** | -.18 | -.35** |

*Note*. ^a^ Four-microstate solution; ^b^ six-microstate solution. * p < .01, ** p < .001

As shown in the above tables, the properties (i.e., spatial topographies and temporal parameters) of resting-state microstate 4 from the optimal, data-driven, six-microstate solution were highly correlated with the properties of resting-state microstate 4 from the four-microstate solution. This was not the case for resting-state microstate 6 (e.g., resting-state microstate 6 is often collapsed with resting-state microstate 3 in solutions with only four microstates; (Custo et al., 2017); as such, the properties of resting-state microstate 4 appear to be uniquely robust to segmentation and may have increased utility relative to the other microstates for comparing effects across multiple timepoints, studies, and samples.

## S22. Full Details of Regression Analyses

* Manuscript does not contain details about step 1 of hierarchical regression analyses; therefore, details are provided here.

*Relationship between residualized ERN amplitude and error-related microstate 3 GEV (model 1; n = 84).* In step one, neither age nor sex explained a significant proportion of the observed variation in error-related microstate 3 GEV, Adj. *R*^2^ = -.01, *F*(2, 81) = 0.71, *p* = .49. In step two, the addition of residualized ERN amplitude to age and sex was associated with a statistically significant increment to the proportion of variation in error-related microstate 3 GEV explained, ∆ Adj. *R*^2^ = .39, *F*(2, 80) = 51.75, *p* < .001, partial adj. *R*^2^ = .39, *f*^2^ = .64, power > .99. The final model containing all three predictors explained a significant proportion of the observed variation in error-related microstate 3 GEV, Adj. *R*^2^ = .38, *F*(3, 80) = 18.02, corrected *p* < .001, *f*^2^ = .61, power > .99. Results indicated that a one standard deviation increase in residualized ERN amplitude associated with a .63 standard deviation decrease in error-related microstate 3 GEV, all else held constant, *t*(80) = -7.18, *p* < .001, 95% CI [-0.05, -0.03] (Figure 3a).

*Relationship between residualized ERN amplitude and resting-state microstate 4 GEV (model 2; n =88).* In step one, age and sex explained a significant proportion of the observed variation in resting-state microstate 4 GEV, Adj. *R*^2^ = .30, *F*(2, 85) = 19.20, *p* = < .001. In step two, the addition of residualized ERN amplitude to age and sex was associated with a statistically significant increment to the proportion of variation in resting-state microstate 4 GEV explained, ∆ Adj. *R*^2^ = .08, *F*(2, 84) = 12.20, *p* < .001, partial adj. *R*^2^ = .12, *f*^2^ = .14, power = .93. The final model containing all three predictors explained a significant proportion of the observed variation in resting-state microstate 4 GEV, Adj. *R*^2^ = .38, *F*(3, 84) = 18.60, corrected *p* < .001, *f*^2^ = .61, power > .99. Results indicated that a one standard deviation increase in residualized ERN amplitude associated with a .30 standard deviation decrease in resting-state microstate 4 GEV, all else held constant, *t*(84) = -3.50, *p* < .001, 95% CI [-0.01, -0.003] (Figure 3b).

*Relationship between error-related microstate 3 GEV and resting-state microstate 4 GEV (model 3; n =82).* In step one, age and sex explained a significant proportion of the observed variation in resting-state microstate 4 GEV, Adj. *R*^2^ = .28, *F*(2, 79) = 16.80, *p* = < .001. In step two, the addition of error-related microstate 3 GEV to age and sex was associated with a statistically significant increment to the proportion of variation in resting-state microstate 4 GEV explained, ∆ Adj. *R*^2^ = .11, *F*(2, 78) = 15.00, *p* < .001, partial adj. *R*^2^ = .15, *f*^2^ = .18, power = .96. The final model containing all three predictors explained a significant proportion of the observed variation in resting-state microstate 4 GEV, Adj. *R*^2^ = .38, *F*(3, 84) = 18.60, corrected *p* < .001, *f*^2^ = .61, power > .99. Results indicated that a one standard deviation increase in error-related microstate 3 GEV associated with a .34 standard deviation increase in resting-state microstate 4 GEV, all else held constant, *t*(78) = 3.88, *p* < .001, 95% CI [0.06, 0.19] (Figure 3c).

*Relationship between resting-state microstate 4 GEV and CBQ-SF effortful control scores (model 4; n = 87).* In step one, sex explained a significant proportion of the observed variation in CBQ effortful control scores, Adj. *R*^2^ = .04, *F*(1, 85) = 4.24, *p* = .04. In step two, the addition of resting-state microstate 4 GEV to sex was not associated with a statistically significant increment to the proportion of variation in CBQ-SF effortful control scores explained, ∆ Adj. *R*^2^ = -.01, *F*(1, 84) = 0.15, *p* = .70. The final model containing all two predictors did not explain a significant proportion of the observed variation in CBQ-SF effortful control scores, Adj. *R*^2^ = .03, *F*(2, 84) = 2.17, corrected *p* = .14. Results indicated that resting-state microstate 4 GEV was not associated with CBQ-SF effortful control, all else held constant, *t*(84) = 0.38, *p* = .70, 95% CI [-4.73, 7.00].

*Relationship between residualized ERN amplitude and PAS scores (model 5; n = 86).* Residualized ERN amplitude did not explain a significant proportion of the observed variation in PAS scores, Adj. *R*^2^ = -.01, *F*(1, 84) = 0.08, corrected *p* = .99.

*Relationship between residualized ERN amplitude and BIS scores (model 6; n = 88).* Residualized ERN amplitude explained a significant proportion of the observed variation in BIS scores, Adj. *R*^2^ = .10, *F*(1, 86) = 10.60, corrected *p* = .003, *f*^2^ = .11, power = .87. Results indicated that a one standard deviation increase in residualized ERN amplitude associated with a .33 standard deviation decrease in BIS scores, *t*(86) = -3.25, *p* = .002, 95% CI [-1.10, -0.27] (plot below).

Scatterplot showing relationship between residualized ERN amplitude and BIS scale.


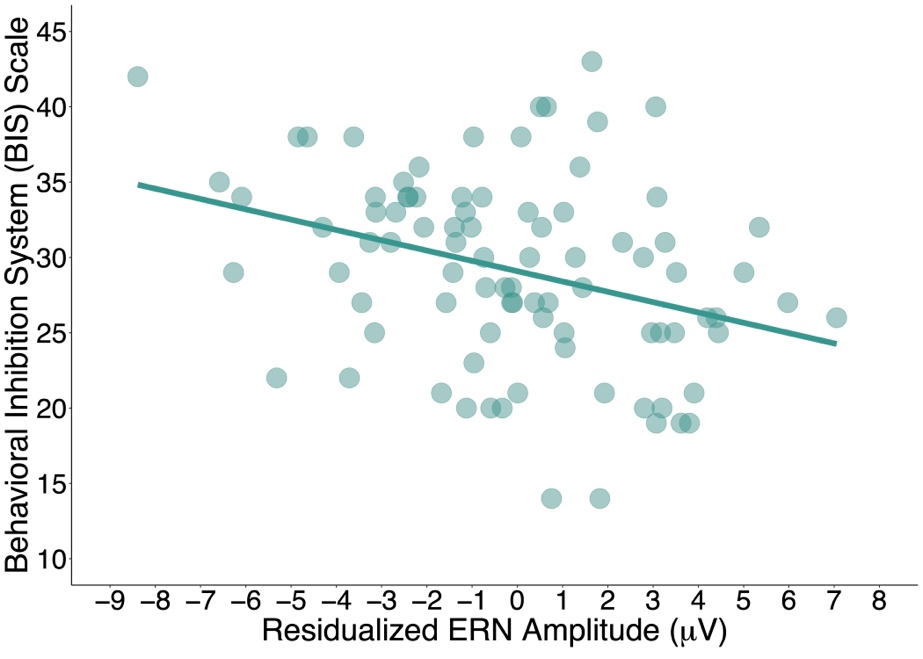


## S23. Results from Models Including Resting-State Microstate 6

*Relationship between residualized ERN amplitude and resting-state microstate 6 GEV (n = 86).* In step one, sex but not age explained a significant proportion of the observed variation in resting-state microstate 6 GEV, Adj. *R*^2^ = .12, *F*(2, 84) = 7.05, *p* < .001. In step two, the addition of residualized ERN amplitude to age and sex was not associated with a statistically significant increment to the proportion of variation in resting-state microstate 6 GEV explained, ∆ Adj. *R*^2^ = -.01, *F*(2, 83) = 0.36, *p* = .55. The final model containing all three predictors explained a significant proportion of the observed variation in resting-state microstate 6 GEV, Adj. *R*^2^ = .12, *F*(3, 83) = 4.78, corrected *p* = .01, *f*^2^ = .14, power = .81. However, results indicated that residualized ERN amplitude was not associated with resting-state microstate 6 GEV, all else held constant, *t*(83) = 0.60, *p* = .55, 95% CI [-0.004, 0.01].

*Relationship between error-related microstate 3 GEV and resting-state microstate 6 GEV (n = 82).* In step one, sex but not age explained a significant proportion of the observed variation in resting-state microstate 6 GEV, Adj. *R*^2^ = .15, *F*(2, 79) = 7.05, *p* < .001. In step two, the addition of error-related microstate 3 GEV to age and sex was not associated with a statistically significant increment to the proportion of variation in resting-state microstate 6 GEV explained, ∆ Adj. *R*^2^ = .01, *F*(2, 78) = 0.01, *p* = .94. The final model containing all three predictors explained a significant proportion of the observed variation in resting-state microstate 6 GEV, Adj. *R*^2^ = .14, *F*(3, 78) = 5.24, corrected *p* = .004, *f*^2^ = .16, power = .86. However, results indicated that error-related microstate 3 GEV was not associated with resting-state microstate 6 GEV, all else held constant, *t*(78) = -0.08, *p* = .94, 95% CI [-0.09, 0.08].

## S24. Neural Sources of Resting-State Microstate 6 and Error-Related Microstate 3

**
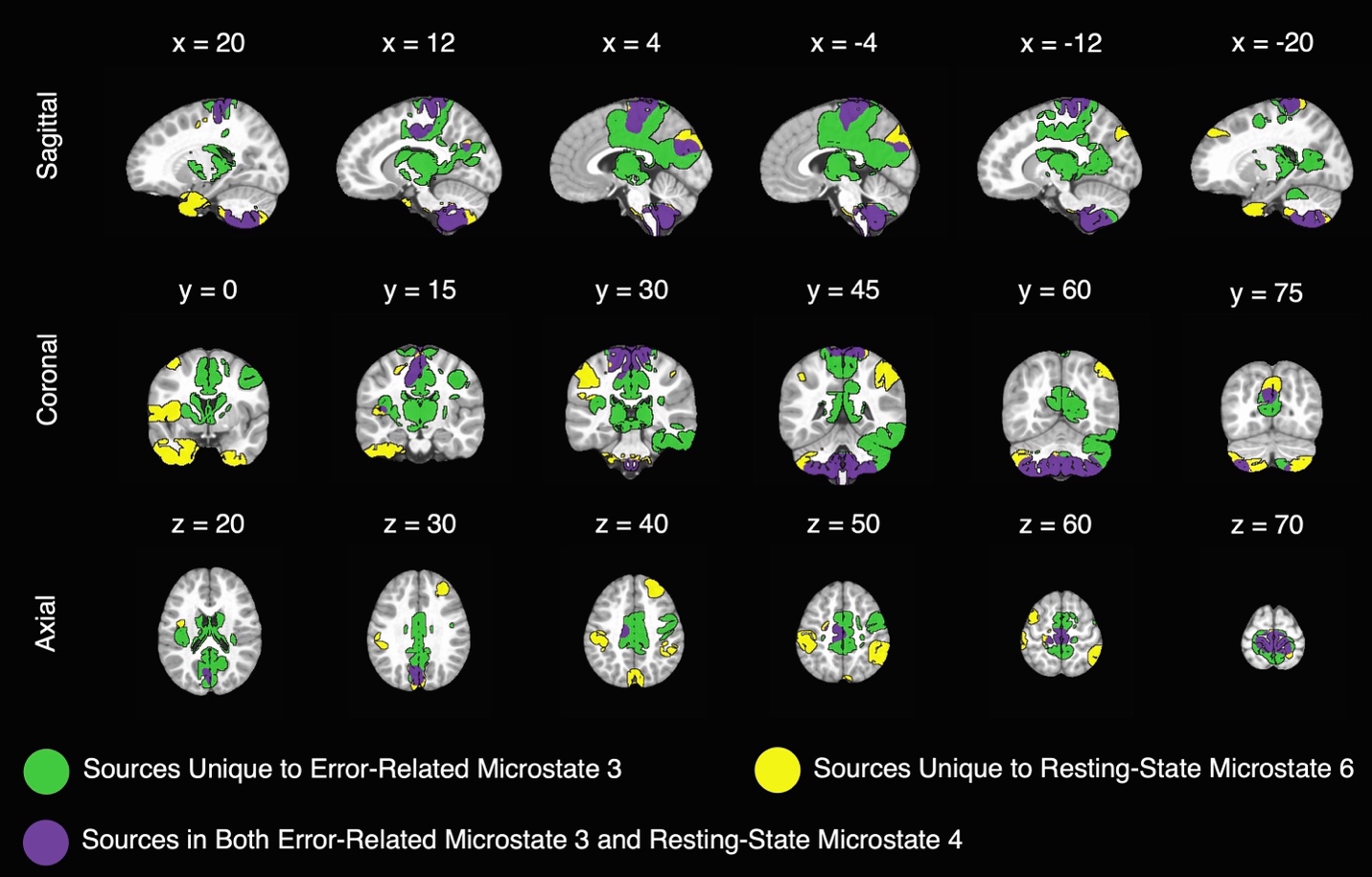
**

*Note.* Sagittal slices (x plane) are presented as left (positive coordinates) to right (negative coordinates) parts of the brain. Coronal slices (y plane) are presented as anterior (negative coordinates) to posterior (positive coordinates) parts of the brain. Axial slices (z plane) are presented as inferior to superior parts of the brain.

## S25. Overlap Between Resting-State Microstate 6 and Error-Related Microstate 3 Sources with Canonical Networks

Percentage of voxels from source localization of microstates that are part of seven canonical functional networks.

| Functional Network | Resting-State Microstate 6 Sources | Error-Related Microstate 3 Sources | Resting-State Microstate 6 Sources + Error-Related Microstate 3 Sources | Overlapping Sources Between Resting-State Microstate 6 & Error-Related Microstate 3 |
| --- | --- | --- | --- | --- |
| Visual | 2.86% | 14.64% | 15.86% | 1.64% |
| Somatomotor | 19.34% | 26.87% | 33.55% | 12.66% |
| Dorsal Attention | 5.50% | 5.57% | 10.71% | 0.36% |
| Ventral Attention | 4.56% | 15.05% | 18.62% | 0.99% |
| Limbic | 22.44% | 1.85% | 24.30% | 0% |
| Control | 9.59% | 8.82% | 17.86% | 0.55% |
| Default | 4.06% | 8.43% | 12.07% | 0.42% |

*Note.* Percentages represent the mathematical intersection of localized microstate sources and functional network sources from the Schaefer-Yeo AFNI 2021 parcellation (7 networks, 100-area). Percentages for each column do not add up to 100% because sources may be located outside of canonical networks.
